# Control system design for a continuous positive airway pressure ventilator

**DOI:** 10.1186/1475-925X-11-5

**Published:** 2012-02-01

**Authors:** Zheng-Long Chen, Zhao-Yan Hu, Hou-De Dai

**Affiliations:** 1 Department of Precise Medical Device, Shanghai Medical Instrumentation College, Shanghai, China; 2 Faculty of Mechanical Engineering, TU Munich, Munich, Germany

## Abstract

Continuous Positive Airway Pressure (CPAP) ventilation remains a mainstay treatment for obstructive sleep apnea syndrome (OSAS). Good pressure stability and pressure reduction during exhalation are of major importance to ensure clinical efficacy and comfort of CPAP therapy. In this study an experimental CPAP ventilator was constructed using an application-specific CPAP blower/motor assembly and a microprocessor. To minimize pressure variations caused by spontaneous breathing as well as the uncomfortable feeling of exhaling against positive pressure, we developed a composite control approach including the feed forward compensator and feedback proportional-integral-derivative (PID) compensator to regulate the pressure delivered to OSAS patients. The Ziegler and Nichols method was used to tune PID controller parameters. And then we used a gas flow analyzer (VT PLUS HF) to test pressure curves, flow curves and pressure-volume loops for the proposed CPAP ventilator. The results showed that it met technical criteria for sleep apnea breathing therapy equipment. Finally, the study made a quantitative comparison of pressure stability between the experimental CPAP ventilator and commercially available CPAP devices.

## Background

Nasal continuous positive airway pressure is a prevalent and effective treatment for patients with obstructive sleep apnea syndrome (OSAS) characterized by repetitive episodes of complete or partial upper airway obstruction that occurs during sleep [1-3]. CPAP devices delivered a positive trans-mural pressure during the throughout respiratory cycle to prevent the collapse of the upper airway. Actually, constant CPAP levels affect the impedance of airway circuit and gas leak, especially the tidal volume and breathing frequency. The key problem to be resolved in designing CPAP devices with good compliance is how to synchronize them with patient's spontaneous breathing, that is, CPAP devices should automatically increase pressure levels at the beginning of inspiration to maintain therapeutic pressure and decrease pressure at the beginning of expiration to facilitate patient's expiration. The present work aims to develop the CPAP ventilator with better pressure stability. The study also tries to come up with some parameters to objectively evaluate the performance of different CPAP devices in an attempt to make CPAP therapy more comfortable and acceptable.

According to British Standards/European Norm/International Organization for Standardization 17510-1:2009 Sleep apnoea breathing therapy-Sleep apnoea breathing therapy equipment, two basic design specifications are

a. Pressure range: 4-20 hPa (4-20 cmH_2_O)

b. Sound levels: ≤30 dB (at 10 hPa)

In order to meet the above design requirements, it is very important to select a sensitive pressure sensor and a motor and blower with good performances such as fast response time and low noise level. In this design, we use an integrated silicon pressure transducer (model MPXV5004GC7U by Freescale Semiconductor, Inc., USA) with pressure range from 0 to 40 hPa and an application-specific CPAP blower (ebm-papst, Germany) that is able to change its speed rapidly to respond to the requirement of the patient. The blower's maximum air flow is 530 ± 10% l/min, maximum back pressure 48 ± 20% cmH_2_O, life expectancy at nominal speed of 30000 r/min more than 20000 h, while the mass is only 0.262 kg. The low noise design offers CPAP patients peaceful and quiet sleep.

## Methods

### A. System Structure

**Figure 1:**
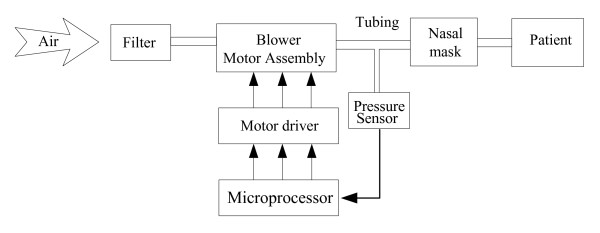
**The block diagram of the experimental CPAP setup**.

The CPAP system consists of a microprocessor, a pressure sensor, a brushless DC motor integrated with a blower, motor controller, flexible tubing and a nasal mask (See Figure 1). Ambient air is drawn through the air filter by the energized blower and is then pressurized. Ultimately, therapeutic pressure is directed to the patient airway via the flexible tubing and a nasal mask. The pressure signal near the nasal mask is detected by the pressure sensor and then fed to the microprocessor. Based on the error between the therapeutic or desired pressure and the measured pressure, the microprocessor regulates the rotational speed by varying the input voltage for the motor driver, which in turn adjusts and controls blower output pressure to keep pressure variations within allowable errors.

### B. Controller Design

**Figure 2:**
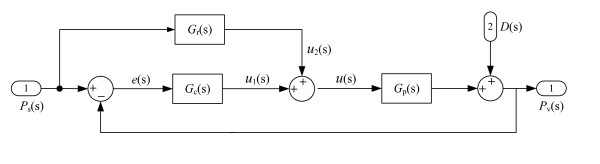
**Control System of the experimental CPAP setup**.

The control scheme of the experimental CPAP system is shown in Figure 2. The composite control system includes a feed forward compensator, *G_r_(s)*, and a feedback controller, *G_c_(s)*, which are designed to achieve the accurate control of therapeutic pressure [4,5]. In Figure 2, *G_p_(s) *represents the transfer function of the motor-blower assembly. *u_1_(s) *and *u_2_(s) *are the output voltage of the feedback controller and that of the feed forward controller respectively. The disturbance caused by patient's breathing is designated as *D(s). P_s_(s) *is the desired (reference) pressure, and *P_v_(s) *signifies the actual pressure.

**Figure 3:**
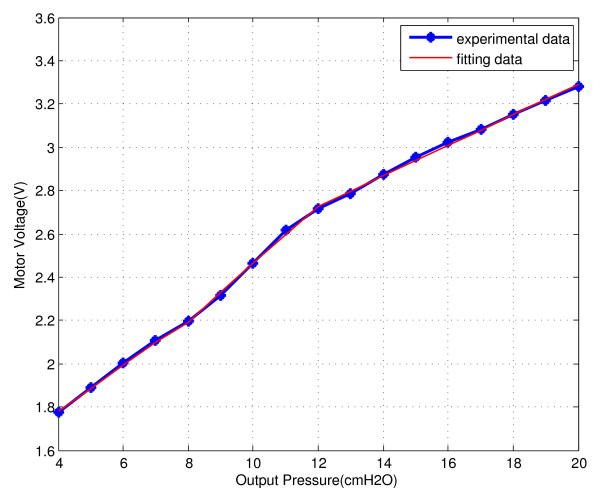
**The relation between motor command voltage and blower output pressure**.

The feed forward controller plays a role in quickly stabilizing the pressure in the nasal mask at the desired set point. In particular, if the user would like to switch over the therapeutic pressure from a high setting to a low one, the feed forward controller can respond with a step signal with large amplitude to the motor driver to achieve its fast acceleration in turn sharp pressure change. That is, the forward feed controller is used to track reference input. To design the forward feed controller, we experimentally determine the relationship between the motor input voltage (*u_2_*) and the blower output pressure (*P*) in open-loop as shown in Figure 3. At this time, the blower outlet is connected to a standard resistance with 4 mm internal diameter. Visually, the relationship of motor input voltage and blower output pressure takes the form of a piecewise linear mapping. A breakpoint around 8 cmH_2_O and another around 12 cmH_2_O divide the graph into three pieces. So we fit the experimental data as the following piecewise linear function:

(1) $$u_{2}\left(P\right)=\left\{\begin{array}{c} 0.1064P+1.3548\text{ if 4}\leq \text{P}<8\\ 0.1342P+1.1204\text{if}8\leq \text{P}<12\\ 0.0705P+1.8813\text{if}12\leq \text{P}\leq \text{20 } \end{array}\right)$$

where *u_2 _*is motor input voltage (unit: volt), and *p *is blower output pressure (unit: cmH_2_O). It can be seen that the error between the model (in red) and the experimental data (in blue) is very small. Therefore we use the piecewise linear function *u_2_(P) *to design the feed forward controller.

On the other hand, the function of the feedback controller is to deal with model uncertainty (such as unmodeled nonlinear dynamics) of blower-motor assembly and to reduce pressure variations caused by patient breath disturbances. We implement a digital PID feedback controller given by [6-8] as follows:

(2) $$G_{c}\left(z\right)=\frac{u_{1}\left(z\right)}{E\left(z\right)}=K_{p}+K_{I}\frac{T\left(z+1\right)}{2\left(z-1\right)}+K_{D}\frac{z-1}{Tz}$$

where T is the sampling period, K_P_, K_I _and K_D _are the gains for proportional, integral and derivative controllers respectively. In practical implementation, the PID controller described by (2) is represented as the following incremental form

(3) $$\begin{array}{ccc} & \Delta u_{1}\left(kT\right)=u_{1}\left(kT\right)-u_{1}\left[\left(k-1\right)T\right] & \\ & =K_{P}e\left(kT\right)+\frac{K_{I}T}{2}\overset{k}{\underset{i=1}{\sum }}\left\{e\left[\left(i-1\right)T\right]+e\left(iT\right)\right\}\\ & +\frac{K_{D}}{T}\left\{e\left(kT\right)-e\left[\left(k-1\right)T\right]\right\}-K_{P}e\left[\left(k-1\right)T\right]\\ & -\frac{K_{I}T}{2}\overset{k-1}{\underset{i=1}{\sum }}\left\{e\left[\left(i-1\right)T\right]+e\left(iT\right)\right\}\\ & -\frac{K_{D}}{T}\left\{e\left[\left(k-1\right)T\right]-e\left[\left(k-2\right)T\right]\right\}\\ & =K_{P}\left\{e\left(kT\right)-e\left[\left(k-1\right)T\right]\right\}\\ & +\frac{K_{I}T}{2}\left\{e\left(kT\right)+e\left[\left(k-1\right)T\right]\right\}\\ & +\frac{K_{D}}{T}\left\{e\left(kT\right)-2e\left[\left(k-1\right)T\right]+e\left[\left(k-2\right)T\right]\right\} \end{array}$$

where k is the sample interval, *K_P_, K_1 _*and *K_D_*, are the proportional, integral and differential gains controller, respectively. Ultimately, the command input voltage *u(S) *of the motor driver is equivalent to *u_1_(S) *plus *u_2_(S)*.

**Figure 4:**
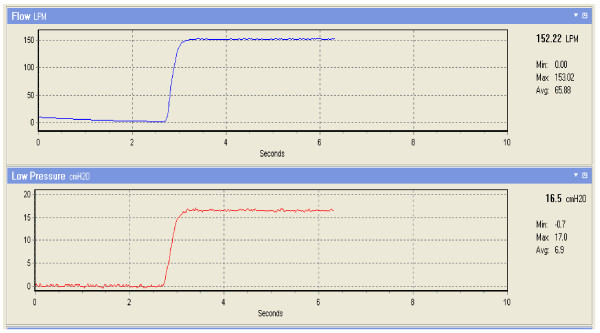
**The flow and pressure response of the experimental CPAP system to step command pressure**.

In order to determine the transfer function of the above PID controller, one needs to compute system parameters via some estimation procedure. Reference [9] established a second-order linear system model for similar CPAP devices and estimated system parameters using a least-squares method. The estimated parameters, however, were not satisfactory due to model errors occurring in the linearization procedure and needed to be refined further. Instead, we heuristically estimate the values of *K_P_, K_J _*and *K_D _*by generating step response and applying the well-known Ziegler and Nichols method (REF). It makes a priori assumption on the system model but does not require that the model be specifically known. Ziegler-Nichols formulae for specifying the controllers are just based on plant step response. Note that the patient is disconnected and the open side of nasal mask is open to atmosphere when the evaluating step response of the system (See Figure 4). PID gains at different pressure levels from 4 cmH_2_O-20 cmH_2_O are listed in the Table 1. The gain K_D _is omitted in Table 1 as it is zero throughout the pressure settings range. The results show that only using the PI controller can obtain the desired performance specifications.

**Table 1:** PI controller parameters at different pressure setting

Pressure Setting (cmH_2_O)	Kp	K_I_	Pressure Setting (cmH_2_O)	Kp	K_I_
4	1.5	0.012	8.5	1.5	0.01
4.5	1.5	0.012	9	1.5	0.01
5	1.5	0.012	9.5	1.5	0.009
5.5	1.5	0.012	10	1.35	0.006
6	1.5	0.012	12	1.35	0.0008
6.5	1.5	0.01	14	1.35	0.0002
7	1.5	0.011	16	1.25	0.0002
7.5	1.5	0.01	18	1.25	0.0002
8	1.5	0.01	20	1.25	0.0002

## Results

**Figure 5:**
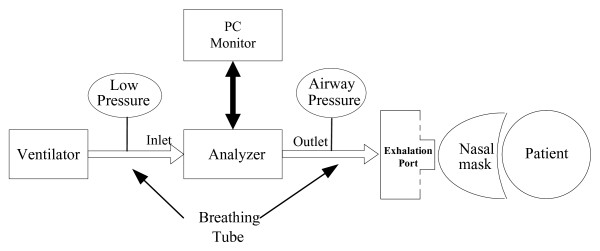
**Test setup for the experimental CPAP ventilator parameters**.

**Figure 6:**
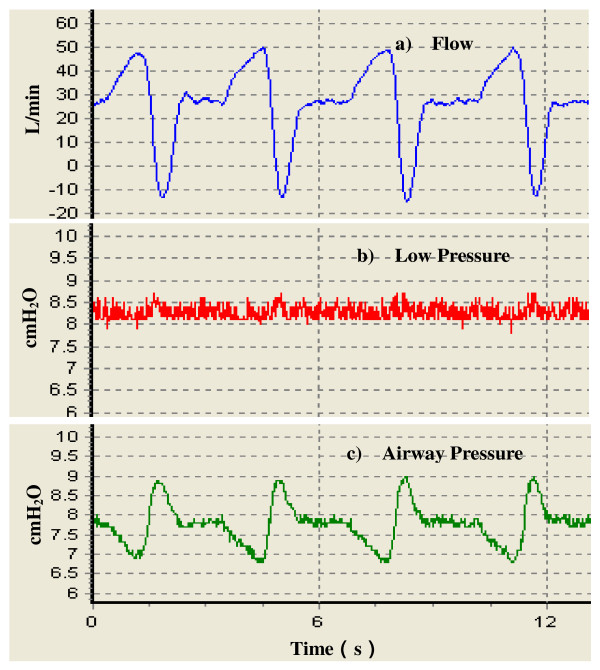
**Original recordings of several breathing waveforms from the experimental CPAP ventilator with PI controller**. a) Flow rate. b) Low pressure. c) Airway pressure. The pressure setting is at 8 cmH_2_O. The maximum inspiratory flow rate is about 50 L/min and the maximum expiratory flow rate about -10 L/min. The output port pressure fluctuation caused by patient's breath is kept within the range of ± 0.5 cmH_2_O.

To validate the efficiency of the proposed control method, gas flow analyzer (VT PLUS HF, Fluke Corporation, USA) is used to test the experimental CPAP ventilator (include in the additional file 1) performance. The test setup (see Figure 5) includes CPAP ventilator, the analyzer, standard breathing hoses, the exhalation port, the nasal mask, the test lung (a volunteer patient) and a PC. First, we connected the gas output port of the ventilator to the flow inlet port of the analyzer using a breathing tube. Then, we connected the flow outlet port of the analyzer to the assembly of the exhalation port and nasal mask using a breathing tube. Finally we applied the nasal mask to the patient. Data calculated by the analyzer is recorded by running the VT for Windows PC software using a PC. The flow and airway pressure waveforms during a few breath cycles are shown in Figure 6. The pressure setting is 8 cmH_2_O. Output port pressure of the ventilator is shown as Low Pressure (middle panel). We can see that it swings within the range of ± 0.5 cmH_2_O, indicating stability. The bottom panel is airway pressure waveform. Peak inspiratory pressure and peak expiratory pressure are about 6.6 cmH_2_O and 9.1 cmH_2_O respectively. Both the most positive and the negative pressure difference from the set value fall within the scope of ± 1.5 cmH_2_O, which meets the U.S. Food and Drug Administration (FDA) and 17510 criteria (± 2 cmH_2_O) for CPAP ventilators [10,11].

**Figure 7:**
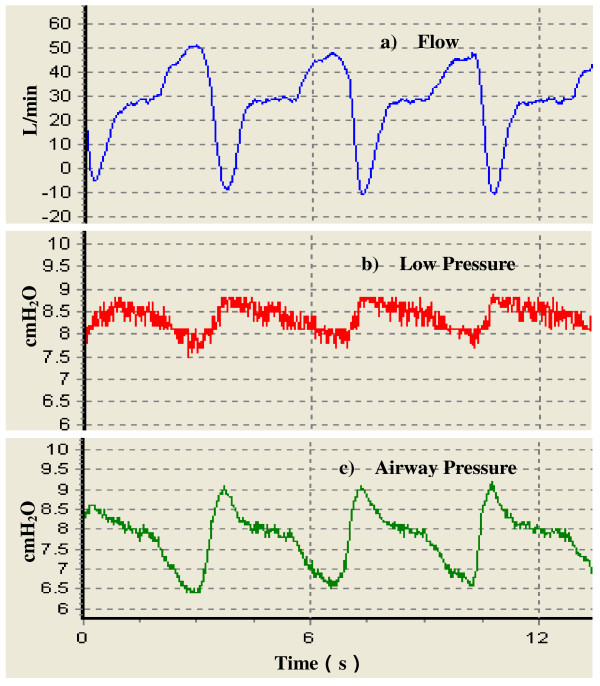
**Original recordings of several breathing waveforms from the experimental CPAP ventilator without PI controller**. a) Flow rate. b) Low pressure. c) Airway pressure. The pressure setting is at 8 cmH_2_O. The maximum inspiratory flow rate is about 50 L/min and the maximum expiratory flow rate about -10 L/min. The output port pressure fluctuation caused by patient's breath is within the range of ± 1 cmH_2_O.

Comparing Figure 6 and 7, for the experimental CPAP ventilator without PI controller operating in open-loop control mode the output port pressure rises approximately 1 cmH_2_O above the set pressure when the maximum expiratory flow rate reaches the same value, approximately -10 L/min. Likewise, with the same maximum inspiratory flow rate about 50 L/min, output port pressure drops about 0.5 cmH_2_O lower than set pressure. This shows that the PI controller improves the transient response and reduces the steady-state error.

It is also observed that in Figure 6 the airway pressure fluctuation is ± 1.5 cmH_2_O while the output port pressure fluctuation is only ± 0.5 cmH_2_O. First, we don't expect the motor speed makes an excessive large or small change to avoid pressure unable to go back to the setpoint before the beginning of the next breathing cycle. Hence, a pressure threshold of 3 cmH_2_O is set in the computer. When the offset between the setpoint and the actual pressure is greater than 3 cmH_2_O the PI controller doesn't work any more. In normal CPAP therapy, the patient is directly connected to the output port. However, in Figure 5 additional breathing tube and the analyzer are included in the patient circuits. In this scenario, resistance of additional breathing tube and the flow sensor (between inlet and outlet of the analyzer and with dynamic resistance < 2 cmH_2_O at 60 lpm) is a non-negligible factor that impedes gas flow and undermines the automatic pressure compensation function played by the CPAP ventilator. Besides, gas leak in the exhalation port also partly weakens the contribution from the PI controller. Taken together, these reasons result in the observed airway pressure fluctuation.

**Figure 8:**
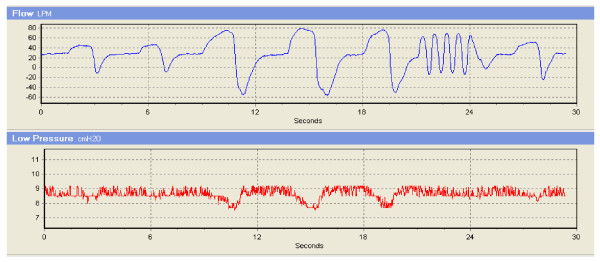
**Original recordings of several breathing waveforms from the experimental CPAP ventilator with PI controller during hyperventilation and high frequency ventilation**.

Quite often, massive flow rate fluctuations occur during apnea and subsequent hyperpnea. Figure 8 shows a few original recordings of the flow rate and pressure from the experimental CPAP device during hyperventilation and high frequency ventilation with the set pressure at 8.5 cmH_2_O. Obviously, the pressure swings are dependent on flow rates during breathing, with pressure drop by approximate 1 cmH_2_O when the max instantaneous inspiratory flow rate (including gas leak) increase from 50 L/min (normal and resting breathing) to 80 L/min (hyperventilation). The pressure is also stable when the max instantaneous expiratory flow rate rises from -10 L/min (normal and resting breathing) to -55 L/min (hyperventilation). In the subsequent high frequency ventilation, pressure variations are relatively small.

**Figure 9:**
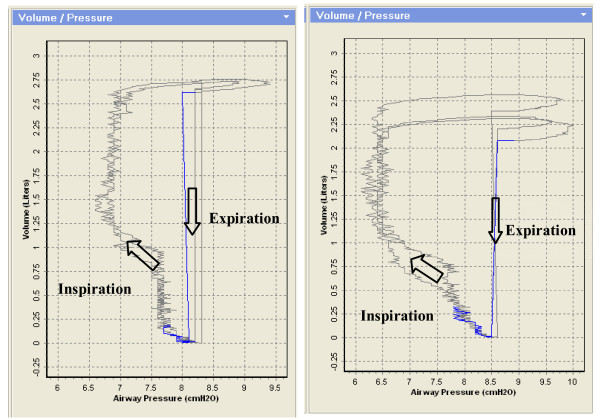
**Pressure-Volume loops from the experimental CPAP ventilator with PI controller (left panel) and without PI controller (right panel)**. The pressure set is at 8.5 cmH_2_O.

Pressure-Volume loops [12,13] from the experimental CPAP ventilator with pressure set at 8.5 cmH_2_O are indicated in Figure 9. The left P-V loops are for CPAP ventilator with PI controller and the right P-V loops for CPAP ventilator without PI controller. The entire area which the loop encompasses represents the patient's work of breathing. In the left panel, the pressure drop during the inspiratory phase is less than that in the right panel due to the pressure compensation from the PI controller. Consequently, the loop area in the left panel is smaller than that in the right panel meaning a patient makes a smaller effort to combat the ventilator's inspiratory resistance. In addition, from the inspiratory limb of P-V loops, it can also be observed that the total volume (including gas leak in the exhalation port) during inspiratory period is different when the pressure is set at the same level. The left panel is approximately 2.75 L and the right panel is 2.25 L during one breathing cycle. That is to say the PI controller can regulate the flow rate according to the patient inspiratory efforts to provide the adequate tidal volume.

## Discussion

The aim of this part is to make a comparison of pressure stability between the experimental CPAP ventilator and commercially available CPAP devices. The test setup is completely similar to that in Figure 5. Flow, volume and pressure are continuously recorded and stored using the VT for Windows PC software.

**Figure 10:**
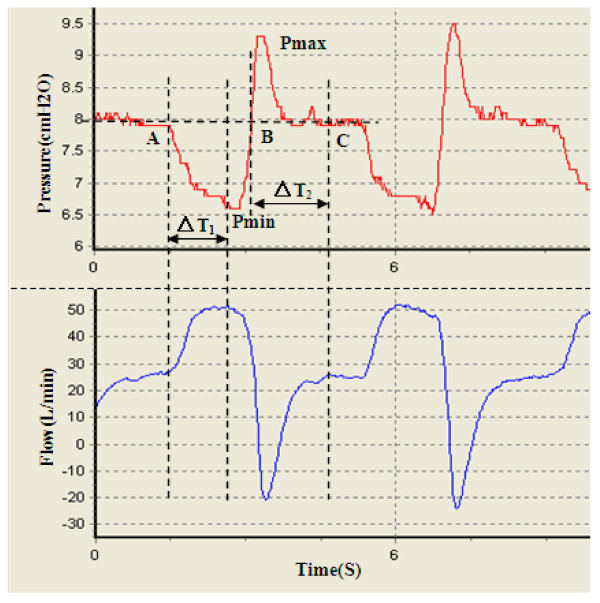
Graphic measurement of minimum inspiratory pressure Pmin, maximum expiratory pressure Pmax, time interval from inspiration onset to Pmin(ΔT1) and time interval during which airway pressure is greater than the pre-set pressure(ΔT2)

**Figure 11:**
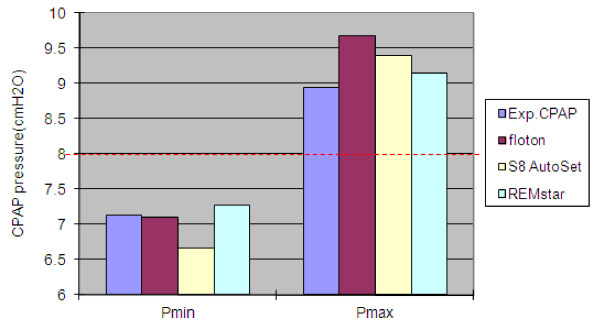
Minimum inspiratory pressure (left) and maximum expiratory pressure (right) in four CPAP devices. The dash line indicates the pre-set pressure 8 cmH_2_O

**Figure 12:**
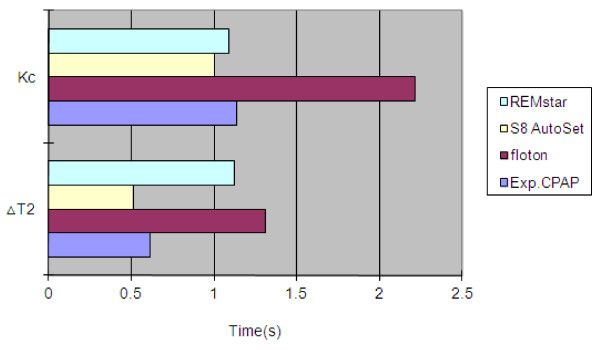
**Kc and ΔT2 in four CPAP devices**. Kc is the ratio of pressure drop during inspiration to time interval ΔT1. ΔT2 is time interval during which airway pressure is greater than the pre-set pressure.

Four devices including the experimental CPAP, floton™(CURATIVE Medical Technology Beijing Ltd, China), S8 AutoSet Spirit (ResMed Ltd, Australia) and REMstar Auto (Respironics, Inc., USA) were compared in the study. We first identified five feature points on the pressure wave during one breath cycle as shown in Figure 10. Pmin and Pmax are the minimum and maximum airway pressure value respectively. Point A is the inspiration onset point. Point B is a transitional point at which the airway pressure equals the pre-set pressure. Point C represents the end expiratory level with the airway pressure dropping down to the pre-set pressure. Kc, the ratio of pressure drop during inspiration to time interval ΔT_1_, reflects the dynamical pressure compensation performance of CPAP devices. The smaller Kc is, the better pressure compensation. ΔT_2 _is the time interval during which airway pressure is greater than the pre-set pressure. Obviously, a shorter time ΔT_2 _reflects more comfortable breathing. Therefore, we select four parameters Pmin, Pmax, Kc and ΔT_2 _to quantitatively compare comfort level across different CPAP devices as shown in Figure 11 and 12. These parameters for each device have been averaged over 10 breaths during visually confirmed steady state. From Figure 11 we can see that pressure swings of our experimental CPAP are smaller than floton™ and S8 AutoSet Spirit. In addition, Figure 12 indicates that the experimental CPAP is superior to floton™ in terms of shorter Δ T_2 _and smaller Kc value.

Another specification that needs to be taken into account is device's noise level. With the output pressure set at 10 cmH_2_O as specified by ISO 17510 we acoustically compare their A-weighted sound power levels and find no significant difference. Further, taking measurements using a sound level meter (TES 1350A, Taiwan), the A-weighted sound power level caused by the experimental CPAP is 44.5 dB and that is 5.8 dB above the A-weighted background level of extraneous noise, 38.7 dB. This device additional noise level is slightly smaller than 6 dB specified by ISO 17510.

After going through an aging test for over four weeks, we evaluated the clinical performance from 6 volunteers. Among them, 2 subjects were OSAS patients. Each subject was tested by standard procedures for 30 minutes. The demographic data and CPAP performance results are shown in Table 2. We can see that the pressure variations during half an hour's CPAP breathing are within the range of ± 2 cmH_2_O for subjects No.3-No.5. And for subject No.1, No.2 and No.6, occasional deep inspiration and expiration cause pressure fluctuation more than 2 cmH_2_O, but in the case of normal and even breathing the pressure fluctuation is still kept within the allowable range of ± 2 cmH_2_O.

**Table 2:** CPAP Clinical Performance Results for 6 Subjects

Subject No.	Remark	Weight (kg)	Height (m)	Age	Sex	Setting Pressure (cmH_2_O)	Max. Pressure (cmH_2_O)	Min. Pressure (cmH_2_O)
1	OSAS patient	101	1.72	43	Male	8.0	10.3	6.2
2	OSAS patient	96	1.70	64	Male	7.0	8.6	4.6

3	Normal subject	60	1.70	29	Male	8.0	9.6	6.2
4	Normal Subject	60	1.78	29	Male	8.0	9.4	6.6
5	Normal subject	58	1.69	26	Male	8.0	9.3	6.1
6	Normal Subject	65	1.68	31	Male	8.0	10.1	6.8

## Conclusions

Using a fast-response blower, pressure transducer and microprocessor, an experimental CPAP ventilator has been built for the treatment for OSAS. A compensation that comprises feed forward control and feedback control integrating PI compensator is proposed to maintain the therapeutic pressure and minimize pressure variations during spontaneous breathing. Comparison of pressure-time curves and P-V loops indicates that a CPAP ventilator with PI pressure feedback control outperforms the ventilator without pressure feedback control.

Taking a close look at Figure 7, one finds when the CPAP pressure is set at 8 cmH_2_O even if the experimental CPAP ventilator operates in the open-loop mode, the output port pressure variations are still less than ± 2 cmH_2_O. Hence, the present study shows that a fast-response motor/blower is the dominant factor determining performance of a CPAP ventilator.

A future study is needed to further eliminate the uncomfortable feeling of exhaling against positive pressure. A feasible solution is to let the controller actively reduce the CPAP pressure during the expiration and then return the setting CPAP pressure before the onset of inspiration. In fact, several commercial CPAP ventilator companies such as Respironics and Resmed have introduced this new technology to their own products [14-16]. To implement this, besides using a pressure sensor, an additional flow sensor is needed to determine the transitional points between exhalation and inhalation. The flow sensor typically has fast response time of 1 to 3 ms, ranges from ± 200 to ± 1000 sccm and features bi-directional sensing capability. It senses the patient's breathing effort by monitoring airflow amount and direction. Then this flow signal is used to control a sleeve valve whose one of the ports is pneumatically connected to the blower pressure and the other is an exhaust outlet. Since the airflow direction is reversed from exhalation to inhalation it is technically easy to identify the breathing phase and to adjust the pressure correspondingly. Another advantage that the flow transducer offers is it makes it possible to compensate flow loss caused by air leaks, which in turn is helpful in maintaining constant CPAP [17,18].

## Competing interests

The authors declare that they have no competing interests.

## Authors' contributions

ZLC conceived, designed and implemented the controller, acquired the data and drafted the manuscript. ZYH supervised the project and research group, and contributed to valuable discussions and suggestions. HDD designed the printed circuit board and carried out the circuit debugging. All authors read and approved the final manuscript.

## Supplementary Material

Additional file 1 **An experimental CPAP ventilator**. A photo of the disassembled CPAP setup Click here for file
