# Supplementary figures and images for: Control system design for a continuous positive airway pressure ventilator
Source: Biomed Eng Online. 2012 Feb 1;11:5. doi: 10.1186/1475-925X-11-5 (PMC3297530; doi:10.1186/1475-925X-11-5)

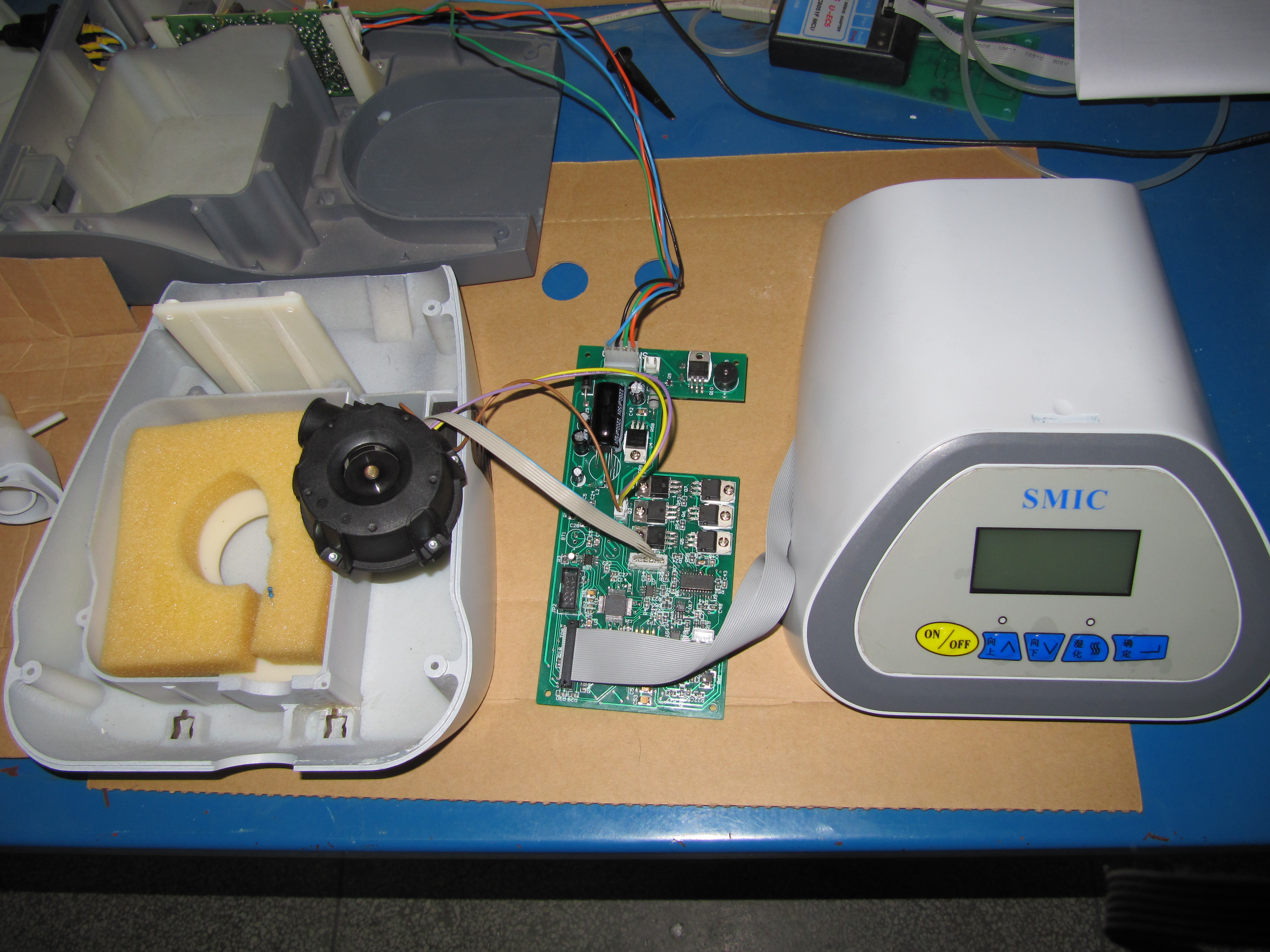

Supplement: Additional file 1 — An experimental CPAP ventilator. A photo of the disassembled CPAP setup [file 1475-925X-11-5-S1.JPEG]
